# Author Correction: Sustainability benefits of transitioning from current diets to plant-based alternatives or whole-food diets in Sweden

**DOI:** 10.1038/s41467-024-47901-5

**Published:** 2024-05-07

**Authors:** Anne Charlotte Bunge, Rachel Mazac, Michael Clark, Amanda Wood, Line Gordon

**Affiliations:** 1 Stockholm Resilience Centre, Stockholm University, Stockholm, Sweden; 2 Helsinki Institute of Sustainability Sciences (HELSUS), University of Helsinki, Helsinki, Finland; 3 Department of Agricultural Sciences, Faculty of Agriculture and Forestry, University of Helsinki, Helsinki, Finland; 4 Smith School of Enterprise and the Environment, Oxford, UK; 5 Department of Biology, University of Oxford, Oxford, UK; 6 Oxford Martin School, University of Oxford, Oxford, UK

**Keywords:** Sustainability, Agriculture

Correction to: *Nature Communications* 10.1038/s41467-024-45328-6, published online 01 February 2024

- The original version of this Article contained an error in sub-section “Nutritional adequacy of the dietary scenarios”, which incorrectly read ‘All scenarios met the recommendation for calcium and riboflavin intake, except the VGNWHOLE diet,’ The correct version deletes ‘and riboflavin’ after ‘calcium’, and adds ‘and flexitarian WF (FLXwhole)’ after GNWHOLE’. This has been corrected in both the PDF and HTML versions of the Article.
- The original version of this Article contained an error in sub-section “Nutritional adequacy of the dietary scenarios”, which incorrectly read ‘B12 decreased to less than recommended levels among the VGNWHOLE, VGNPBA and vegetarian WFs (VGTWHOLE) scenarios.’ The correct version replaces this sentence with ‘B12 decreased to less than recommended levels among the VGNWHOLE and VGNPBA scenarios.’. This has been corrected in both the PDF and HTML versions of the Article.
- The original version of this Article contained an error in sub-section “Nutritional adequacy of the dietary scenarios”, which incorrectly read ‘All scenarios including the BAU met the thiamine, vitamin E, C, and B6 recommendations,’ The correct version replaces this sentence with ‘All scenarios including the BAU met the iron, magnesium, phosphorus, vitamin E and C recommendations,’. This has been corrected in both the PDF and HTML versions of the Article.
- The original version of this Article contained errors in the plotting of in Fig. 1 towards the values of the X-axis and variables. This has been corrected in both the PDF and HTML versions of the Article.
- The original version of the Supplementary Information associated with this Article contained an error in the plotting of Supplementary Fig. 3 towards the value of the X-axis and the variables. The HTML has been updated to include a corrected version of the Supplementary Information.

