# Supplementary material for: Sustainability benefits of transitioning from current diets to plant-based alternatives or whole-food diets in Sweden
Source: Nat Commun. 2024 Feb 1;15:951. doi: 10.1038/s41467-024-45328-6 (PMC10831109; doi:10.1038/s41467-024-45328-6)
Supplement: Supplementary file 1 — Supplementary Information [file 41467_2024_45328_MOESM1_ESM.pdf]

Supplementary Information for

Sustainability benefits of transitioning from current diets to plant-based alternatives or whole-food diets in Sweden

Author list

Anne Charlotte Bunge[1] *(Corresponding author, annecharlotte.bunge@su.se)*

Rachel Mazac[1,2,3]

Michael Clark[4,5]

Amanda Wood[1]

Line J. Gordon[1]

Affiliations

1 Stockholm Resilience Centre, Stockholm University, Stockholm, Sweden

2 Helsinki Institute of Sustainability Sciences (HELSUS), University of Helsinki, Helsinki, Finland

3 Department of Agricultural Sciences, Faculty of Agriculture and Forestry, University of Helsinki, Helsinki, Finland

4 Smith School of Enterprise and the Environment, Oxford, United Kingdom

5 Department of Biology, University of Oxford, Oxford, United Kingdom

Table of Contents

A.1 Supplementary methods 3
A.1.1 Designing the dietary scenarios 3
Designing the business-as-usual (BAU) diet 3
Composing the alternative dietary scenarios 4
A.1.2 Nutrient Analysis 7
A.1.3 Environmental Impact Analysis 7
A.1.4 Daily Food Expenditure Analysis 8
A.2 Supplementary results 9
A.2.1 Detailed Results from the nutritional performance analysis 9
A.2.2 Results from the uncertainty analysis 11
References 16

A.1 Supplementary Methods

Supplementary Figure 1. Overview of the methodology used to design the respective dietary scenarios and for the combined assessment of environmental impact, nutritional adequacy and daily food expenditure of these diets

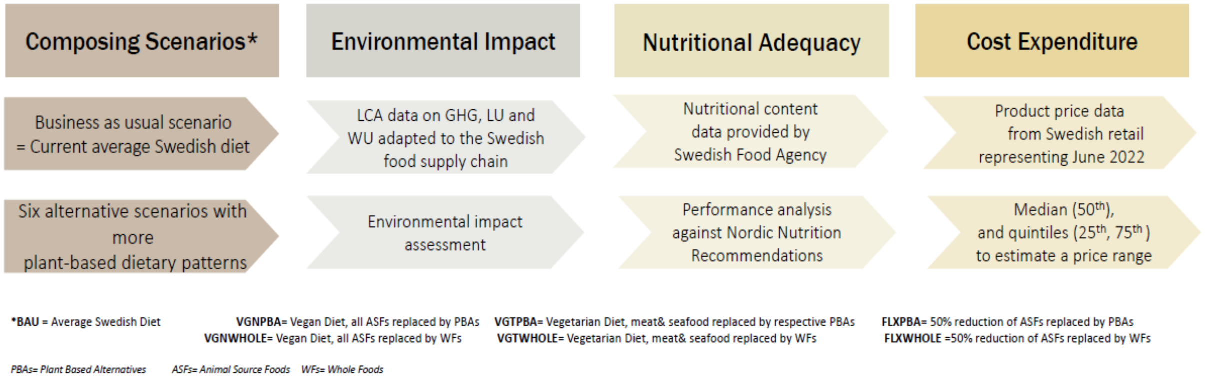

A.1.1 Designing the dietary scenarios

Designing the business-as-usual (BAU) diet

We derived a current average Swedish diet and six dietary scenarios to assess the environmental, nutritional, and economic implications of shifting towards diets high in PBAs or WFs.

To compose the current average diet (hereafter BAU), we derived data from the Swedish Statistical Database (Swedish Board of Agriculture).[1] Data for the direct consumption of food items in kg or L/ per capita and year was obtained and divided by a factor of 365 in order to estimate daily food consumption per capita. The per capita approach divides the quantity of food items available by the total population that lived in the country in the year 2019. The data represents the average food supply that is purchased by the population as a whole. As such it includes the amount of food available for consumption in Swedish households and institutional kitchens. The data does not account for food loss and waste generated at the household stage. Hence, we applied waste ratios as provided by the FAO study "Global Food Losses and Food Waste - extent, causes, and prevention" to account for waste generated at the household stage (Table 1)[2]. These waste ratios have been applied by previous dietary modelling studies and therefore enable comparison and consistency[3,4] .

Supplementary Table 1. Ratio of waste generated at the household level. Based on waste ratios provided by the Food and Agricultural Organization (2011).

Food Group	Waste At Household Stage
Cereals	25%
Roots and Tubers	17%
Oilseeds and pulses	4%
FRUIT AND VEGETABLES	19%
MEAT	11%
SEAFOOD	11%
MILK	7%
BEVERAGES, OTHER	7%

For the purpose of our study, we focus on food products and non-alcoholic beverages. We exclude alcoholic drinks, tea, broth, syrup, gruel powder, potato starch, powder for mashed potatoes or turnips, canned, chilled and deep-frozen potato products (only focus on fresh potatoes to ensure that they match the nutritional values) and baby and toddler nutrition to focus on the products that are of relevance to our study.

For the food groups where the Swedish Statistical Database did not provide information on average consumption, we supplemented the dataset with data from other sources. For example, data on the consumption of seafood was

limited to canned herring, caviar and other products of roe, prepared or canned crustaceans and molluscs, and processed fish such as fish fingers with no data provided on the consumption of fresh fish. We, therefore, obtained data on seafood consumption from a report provided by the RISE Research Institute titled Swedish Consumption of Seafood[5]. According to the report, an average of 12kg per person was consumed in the year 2019, corresponding to 230 grams per week. Divided by a factor of seven, this resulted in daily consumption of 32.6 g/capita/day. After applying the FAO waste ratios this resulted in 29.4g/capita/day. The five most commonly consumed seafood products in Sweden are salmon, herring, cod, North Sea prawns, and unspecified prawns. Hence, we focused on and calculated medians of the nutritional values of the respective food groups. Although seafood is a diverse group that contains both animals and plants (e.g. algae), we here focused on living animals.

The Swedish Statistical Database does not provide separate information on the consumption of legumes and soy foods. The consumption of legumes is categorised under 'root vegetables and other vegetables, frozen or dried' and 'vegetables prepared or preserved otherwise than by vinegar or acetic acid". In general, data on the consumption of legumes in Sweden is rarely available, and neither is there a specific recommendation on the intake provided in the Swedish dietary guidelines. The most recent available data on per capita consumption of legumes in Sweden is considered to be the results of the Riksmaten food survey corresponding to consumption patterns among adults for the years 2010/2011. The data stated that the daily per capita consumption of legumes in Sweden was 12 g, but that eating patterns differ considerably between individuals and only 50% of Swedish women and 44% of Swedish men include legumes in their diet (NFA, 2012)[6]. Hence, and in line with the available dietary intake data from the Swedish Statistical Database, we decided to compose the BAU diet without separated intakes of legumes and fermented legumes such as tofu. The database further does not provide information on the intake of PBAs, we thus set the intake of PBAs to zero in the BAU diet.

Methodological validation

To assess the validity of using the Swedish Statistical Database (Jordbruksverket) for the dietary consumption data, we compared our initial results with existing literature. For the BAU diet, we found an average daily intake of 2450 kcal, the other scenarios were in the range of 2470-2875 kcals. For comparison, the EAT-Lancet study uses a reference intake of 2500 kcal/day for a healthy diet—they state that consuming 2500 kcal per day corresponds to the average energy needs of a 70-kg man aged 30 years and a 60-kg woman aged 30 years whose level of physical activity is moderate to high[7]. Additionally, according to the Swedish National Food Agency, 2500 kcal/day is the upper recommendation for younger women and the lower to median recommendation for men, depending on their age and physical activity level[8]. This revealed that our scenarios and data adjustments for waste and food loss were well within a healthy range for the energy intake of Swedish adults. While it is an average for the whole society, Jordbruksverket does not provide stratified data for different age or sex groups. We recognise that, even if we follow standard practices to adapt dietary guidelines to different population groups, this is a simplification that particularly overestimates consumption for children and elderly, while potentially underestimating consumption for very physically active adults.

Composing the alternative dietary scenarios

We composed six scenarios that reflect more plant-based diets (Tables 2 and 3) using two different functional units (see below). These included vegan scenarios where all ASFs are replaced by PBAs (VGNPBA) or whole foods (VGNWHOLE); vegetarian scenarios where meat and seafood products are replaced by PBAs (VGTPBA) or whole foods (VGTWHOLE), and flexitarian scenarios where 50% of ASFs are replaced with PBAs (FLXPBA) or whole foods (FLXWHOLE).

We did not include what is referred to as novel foods in the European Union, such as insects or cellular meat, in our scenarios as we focus on foods that are already available on the current Swedish food supply chain. We classified tofu and tempeh into the category of legumes as they are traditional products that are not intended to mimic meat products but instead have been traditional protein sources in various parts of the world.

We decided to construct and analyse the scenarios both on a mass and energy basis. Previous studies affirmed that choosing the functional unit has a decisive impact on the environmental, nutritional and economic performance of food items.[9,10] For example, assessing the environmental impact on a mass basis comparison favours energy-dense foods and disadvantages foods with high water content such as vegetables. The opposite applies when

assessing foods on an energy basis. Hence, analysing the scenarios on both functional units strengthens the comparison and accounts for the potential advantages and disadvantages of the different functional units.

Decision to base the scenario on mass basis: We chose to replace food items based on a mass unit (g or ml) as we assumed that, when shopping for foods, consumers base their purchase decisions on the amount of food, rather than the energy and protein content. For example, we assume that people would replace cow milk with the same amount of plant-based milk or meat mince with the same amount of mince based on soy or peas. This is in line with previous studies that conducted similar analyses[11]**.** Each of the dietary scenarios provided a total of 1700 (±50) grams of food per day.

Decision to conduct the scenarios also on an energy basis: We chose to replace based on an energy (kcal) content basis as this comprises both the fat, protein and carbohydrates transitions and thereby allows for a broader investigation for macro- and micronutrient adequacy. To ensure that all scenarios provide the same amount of energy (kcal) we used the BAU diet as the baseline and derived the different scenarios by using the same amount of kcal in the respective food groups. Each of the dietary scenarios including the BAU diet provided a total of 2450 kcal per day.

Supplementary Figure 2. Distribution of food categories in the current diet and respective dietary scenarios. Replacements of animal-source foods from the business-as-usual (BAU) current diet with whole foods (VGTWHOLE, VGNWHOLE, FLXWHOLE) or plant-based alternatives (FLXALT, VGTALT, VGNALT) were made on a mass (a) and (b) energy basis. **BAU** = Average Swedish Diet; **VGNPBA**= Vegan Diet, all ASFs replaced by PBAs; **VGTPBA**= Vegetarian Diet, meat& seafood replaced by respective PBAs; **FLXPBA**= 50% reduction of ASFs replaced by PBAs; **VGNWHOLE**= Vegan Diet, all ASFs replaced by WFs; **VGTWHOLE**= Vegetarian Diet, meat& seafood replaced by WFs; **FLXWHOLE** =50% reduction of ASFs replaced by WFs. The category "other" includes sugar and sugar-based products, salt, coffee, soft drinks, and juice. The acronym PB in the food category means "plant-based". Source data are provided as a Source Data file.

Figure 2a: based on a mass-basis

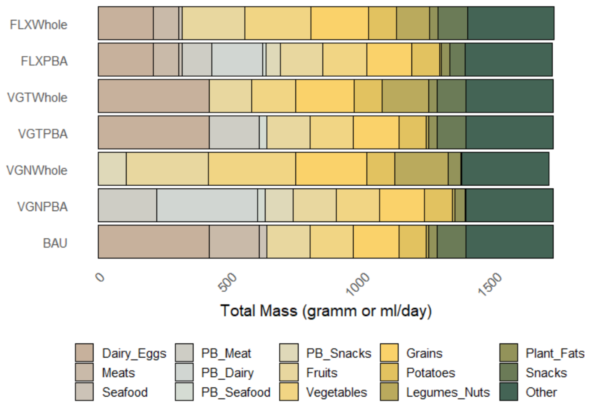

Figure 2b: based on an energy basis

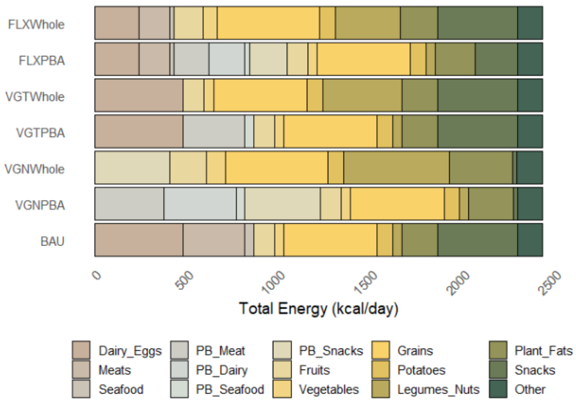

Supplementary Table 2: Composing the dietary scenarios based on a mass and energy functional unit

Decisions to compose the scenarios		
**Scenario**	**Mass Basis (g or ml/day)**	**Energy Basis (kcal/day)**
**VGNPBA**	The amount of consumed ASFs in the BAU diet has been replaced with their respective PBAs (Meat, seafood, dairy products, snacks), without altering the consumption of any other foods (i.e. not modifying the consumption of grains or vegetables, etc.). Hence, we set the consumption of all ASFs to zero. Plant-based egg alternatives are currently not available on the Swedish market, hence we decided to move the replacement of the amount of eggs consumed per day (30.4 g) to PB meat alternatives.	The amount of kcal consumed in the BAU diet from ASFs (780.9 kcal) has been allocated to their respective PBAs (meat, seafood, dairy products, snacks), without altering the consumption of any other foods (i.e. not modifying the consumption of grains or vegetables, etc.). The amount of kcal from eggs (42.3) was allocated to PB meat alternatives.
**VGNWHOLE**	The consumption of ASFs in the BAU diet has been replaced with whole foods such as legumes, grains and vegetables. To allocate the mass of food products from the ASFs in the BAU diet (627.3g), we followed the healthy reference diet as proposed by the EAT-Lancet Commission.[7] We used the proposed upper reference values for vegetables, fruits, legumes, soy foods, nuts, and grains. Hence, we increased the amount of consumed nuts from 7.9 - >50g/day; the amount of legumes from 0-> 100g/day; and soy foods 0->50g/day. For vegetables and fruits, we doubled the amount of consumption as in the BAU diet, equally for all food items. This increased the consumption of vegetables from 164.5g/day->329 g/day and fruits from 148.4 g/day ->296.8 g/day. We increased the consumption of unsaturated oil from 5->15g/day and added 100g of whole grains such as bulgur wheat. The EAT-Lancet healthy reference was used for all whole food scenarios.	The amount of kcal consumed in the BAU diet from ASFs (780.9 kcal) has been distributed to grains, vegetables, legumes, soy foods, unsaturated oils and nuts following the healthy eating guidelines provided by the EAT-Lancet Commission.[10] We doubled the amount of current kcal consumed from fruits and vegetables, increasing the consumption of legumes (172 kcal/day); soy foods (112 kcal/ day); nuts from 47.8 to 290 kcal/day; unsaturated oil 44.2 to 144.2 kcal/day and added 100 kcal from wholegrains such as bulgur. The EAT-Lancet healthy reference was used for all whole food scenarios.
**VGTPBA**	The amount of meat and seafood consumed in the BAU diet was allocated to their respective PBAs, without altering the consumption of any other foods.	The kcals consumed from meat and seafood in the BAU diet were allocated to their respective PBAs, without altering the consumption of any other foods.
**VGTWHOLE**	The amount of current meat and seafood consumption has been replaced with legumes, soy foods, grains and nuts. We chose the same amount of legumes (100g) and soy foods (50g) as in the VGNWHOLE scenario and increased the amount of grains by 50g and of nuts by 20g.	The amount of kcals consumed by meat and seafood products in the BAU diet has been allocated to legumes (172 kcal); soy foods (112 kcal) and nuts (increase from 47.8 to 145.8 kcal.)
**FLXPBA**	We here assume that individuals occasionally shift to PBAs to reduce their consumption of ASFs but remain consuming them on a reduced basis. We chose a 50% reduction scenario in line with current evidence that suggests that a 50% reduction from current levels of ASFs is required.[12] Hence, equal consumption of ASFs and PBAs was assumed, making it the scenario with the highest variety of consumed food items.	The amount of kcal consumed from ASFs in the BAU diet was evenly distributed between ASFs and their respective PBAs, making it the scenario with the greatest diversity of products consumed
**FLXWHOLE**	We here assume that individuals in general reduced their ASFs and upscaled their consumption of legumes, grains and vegetables instead. Hence, equal consumption of ASFs and whole foods was assumed. We increased the consumption of legumes (50g), soy foods (25g), nuts (17g), unsaturated oil (10g), wholegrain (50g) and fruits and vegetables by a factor of 1.5 equally for all food items.	Assuming that individuals in general reduced their consumption of ASFs and upscaled their consumption of legumes, grains and nuts. Hence, equal consumption of ASFs and whole foods was assumed. We increased the consumption of legumes (86kcal), soy foods (56kcal), and nuts (73 kcal).

A.1.2 Nutrient Analysis

To assess the nutritional quality of the respective dietary scenarios, we paired each of the included food items with respective data on their nutritional composition. Nutritional values per kg or litre of food product were derived from the Swedish National Food Agency (*livsmedelsdatabas version 2022-05-24*).[13] The database provides information on the nutritional composition of 55 macro-and micronutrients for more than 2000 foods and dishes available in the Swedish food supply chain. From the retrieved data sheet, we selected the food items that have been relevant for the purpose of this study. Hence, we excluded meals and the food categories that were not of relevance (i.e. flavouring and herbs, alcoholic drinks, nutrient supply, baby and toddler foods etc).

To ensure that we represent the nutritional values of the food products at the consumer pre-cooking stage, we included nutrient values for the food item as most unprocessed. For example, for pasta, we used the median nutritional values for dried wheat and wholegrain pasta instead of boiled pasta without salt, pasta boiled with salt, filled tortellini, or egg pasta to match the food items as included in the dietary scenarios. The same accounts for wholegrains such as couscous, bulgur or rice and legumes where we also used the nutritional values for dried and raw grains and legumes. This aligns with the included food items in the dietary scenarios. For meat and seafood products, we excluded all food items that included "fried"; "boiled w/ salt"; "cooked with gravy" to ensure that we receive nutritional values for the food product as mostly unprocessed.

The database did not provide data on the nutritional ingredients of plant-based seafood alternatives, except for seaweed caviar (*tångcaviar*)- which is not a good representative of seafood alternatives since the general consumption of "caviar and other products of roe" was found to be minimal in comparison to other seafood.[5] Hence, we supplemented the database with nutritional data of plant-based salmon and tuna alternatives that are available at the Swedish food retail market.[14] For the PBA snacks, we selected representative food items from the nutritional values database. For PBA chocolate, we used dark chocolate >70%; for PBA biscuits we used digestive cookies (23% wholegrain); for buns and pastries we used frozen cinnamon buns based on rapeseed oil and for PBA ice cream we used oat-based ice cream.

A.1.3 Environmental Impact Analysis

For our environmental analysis, we used available life cycle assessment (LCA) data and harmonised it into one inventory database (see supplemental data).

Environmental impact data of food items consumed in the BAU diet were sourced from Moberg et al. (2020)[15]. The authors calculated the environmental impacts caused by the average Swedish diet by first assessing the environmental impact per kg or litre of food and then multiplying it by the amounts of food products purchased during one year. The data provides calculated weighted averages of the food items based on production location and imported, as consumed in Sweden. Since the study provides the environmental impact data for food items consumed in Sweden according to the Swedish Board of Agriculture, no environmental impact data is available on products that have not been included in the database (i.e. PBAs, legumes, soy foods).

We, therefore, obtained data for legumes, soy foods, PB meat and dairy alternatives from the report "Environmental impact of plant-based foods–data collection for the development of a consumer guide for plant-based foods in Sweden."[16,17]. The report was prepared by researchers from the Swedish University of Agricultural Sciences for WWF Sweden to provide scientific background information for its consumer guide on plant-based products targeting Swedish consumers. This "*Vego-guide*" aims to target Swedish consumers and therefore contains information on food products that are especially relevant to the Swedish food supply chain. It is based on a literature review that compiled life cycle assessment (LCA) studies on 90 products, from 123 scientific papers, 31 conference papers, 42 reports, and other grey literature, as well as data from two LCA databases. Environmental impact data of products were included for countries of origin that contribute to more than 10% of total imports of the respective food commodity. The report provides ranges for the environmental impact of food items based on the results of the included studies, which, for example, can be attributed to the different countries of origin. Products produced in Sweden almost always were found to have a lower environmental impact than when produced in other countries. However, since many of the legumes consumed in Sweden are imported[18], we used averages across all countries of origin instead of Sweden as the country of origin. We calculated mean values

for the main analysis and conducted a sensitivity analysis with the upper and lower boundaries. When the authors stated that the data should be considered uncertain, for example, because the results could not be validated or/and just one study was found, the data was not included. We used data for oat drink as a PB dairy alternative; oat cream for PB cream alternative; and calculated average values for Quorn and soy-based products as PB meat alternatives. The functional unit is 1 kg edible product in a store in Sweden to enable a fair comparison between canned beans, dry beans, and ready-made protein sources (such as PBAs). Hence, the authors adjusted the environmental impact data of dry legumes to be comparable with canned legumes and ready-to-eat alternatives. For this purpose, specific conversion factors were applied, which were calculated based on the protein content of dry and cooked beans. This included adding the cooking process to the dried legumes as this is included in canned legumes. The report provides a comprehensive set of appendices, providing examples of data from all underlying studies for all individual products and specific details on the methodology used in the underlying studies[16].

For those PBAs, where the report did not provide environmental impact data (i.e., plant-based cheese, seafood and ice cream), we used data provided by foodDB. foodDB is a food composition database which collects information on food products sold at major food retailers in the UK and Ireland[19]. This information was then used to estimate the environmental impacts of these food products across multiple environmental indicators (see Clark et al 2022)[20].

A.1.4 Daily Food Expenditure Analysis

For our analysis, we used prices for ~1000 food items available at the Swedish food supply chain, covering 78 food items aggregated into 15 food groups that we used to construct the scenarios. We extracted data from the discount retailer *Willy´s*, which is part of the leading food retail group *Axfood*. The retailer Willys has the same price values in all physical stores across Sweden and also the same prices in e-commerce, contrary to other retailers that adapt prices to the size and location of the retail store.[21] The data represents market price values in June 2022. We extracted food prices for those food items that have been relevant for the purpose of this study. Hence, we did not extract price data for ready-to eat meals and food categories that were not of relevance (i.e. flavouring and herbs, alcoholic drinks, nutrient supply, baby and toddler foods etc). If food products would fit under several food items, we allocated it to the best suitable one. For example, we allocated prices of a pork sausage to the food item "Cured meat and provisions including sausages" instead of to the food item "Meats, Pork, fresh and frozen". We then calculated median, 25th and 75th percentiles as well as maximum and minimum values for each food item (n=78). We used median values for the main analysis and percentile ranges for the uncertainty analysis.

A.2 Supplementary results

A.2.1 Detailed Results from the nutritional performance analysis

Table 3 and 4 provide an overview of how the different dietary scenarios align with the daily dietary recommendations for macro-and micro nutrients from the Nordic Nutrition Recommendations (NNR)[22] and European Food Safety Authority (EFSA)[23], both when assessed using a mass based and energy based functional unit.

Supplementary Table 3. Nutritional performance of the dietary scenarios in alignment with the Recommended Dietary Allowances by the Nordic Nutrition Recommendations – FUNCTIONAL UNIT: MASS BASIS

**Nutrient**	**Recommended**	**Diet Scenario**						
		**BAU**	**VGNPBA**	**VGNWHOLE**	**VGTPBA**	**VGTWHOLE**	**FLXPBA**	**FLXWHOLE**
Calcium, mg	>= 800·0	956·0	780·2	456·3	1084·3	1121·6	868·3	754·0
Fat total, g	= 65·3	102·5	103·9	99·2	104·5	96·0	100·6	89·3
Fibre, g	>=25	19·4	33·3	50·9	28·5	39·5	26·3	38·3
Folate, ug	>=300	261·7	329·9	678·7	380·9	654·6	295·8	561·1
Iron, mg	>=9	9·9	16·5	24·5	11·4	16·5	13·2	16·2
Magnesium, mg	>=280	296·5	372·3	732·7	355·2	586·7	334·4	520·5
Phosphorus, mg	>=600	1 580·4	1 183·0	1 598·6	1 535·5	1 880·4	1382·1	1 673·3
Potassium, mg	>=3100	2 931·4	2 842·5	4 157·5	2 978·3	3 749·2	2887·5	3 748·0
Protein, g	=58·75	95·2	74·2	75·4	89·9	87·7	84·7	89·5
Riboflavin, mg	>=1·2	1·7	1·3	0·8	1·5	1·6	1·5	1·3
Selenium, ug	>=50	47·5	24·3	17·9	38·1	30·1	35·9	32·6
Sodium, mg	<=2400	2 803·4	3 466·9	2 069·2	3 298·7	2 316·9	3 119·4	2 403·3
Monounsaturated fatty acids, g	<=115	34·1	36·9	33·3	34·1	30·1	34·5	30·0
Polyunsaturated fatty acids, g	>=13	13·7	23·1	26·5	19·0	17·8	18·0	17·3
Polyunsaturated fatty acids, g	<=26·1	13·7	23·1	26·5	19·0	17·8	18·0	17·3
Saturated fatty acids, g	<=26	42·2	27·7	26·2	36·7	36·3	34·0	31·0
Thiamine, mg	>=1·1	1·4	1·0	1·8	1·2	1·6	1·2	1·7
Vitamin B12, ug	>=2	5·7	1·2	0·0	2·9	2·9	3·5	3·0
Vitamin B6, mg	>=1·2	1·9	1·1	2·3	1·3	2·0	1·5	2·2
Vitamin C, mg	>=75	84·4	83·8	141·8	84·5	84·3	84·1	113·1
Vitamin D, ug	>=10	11·1	10·0	6·6	8·4	8·4	9·9	8·4
Vitamin E, mg	>=8	11·3	19·0	12·3	18·1	10·9	14·8	10·6
Vitamin K, ug	>=70	113·3	90·5	607·0	95·7	575·6	99·9	478·6
Zinc, mg	>=7	47·5	23·4	17·0	38·1	30·1	35·5	32·6

BAU *= Average Swedish Diet;* ***VGNPBA****= Vegan Diet, all ASFs replaced by PBAs;* ***VGTPBA****= Vegetarian Diet, meat& seafood replaced by respective PBAs;* ***FLXPBA****= 50% reduction of ASFs replaced by PBAs;* ***VGNWHOLE****= Vegan Diet, all ASFs replaced by WFs;* ***VGTWHOLE****= Vegetarian Diet, meat& seafood replaced by WFs;* ***FLXWHOLE*** *=50% reduction of ASFs replaced by WFs.*

Supplemental Table 4. Nutritional performance of the dietary scenarios in alignment with the Recommended Dietary Allowances by the Nordic Nutrition Recommendations – FUNCTIONAL UNIT: ENERGY BASIS

Nutrient	Recommended	Diet Scenario						
		BAU	VGNPBA	VGNWHOLE	VGTPBA	VGTWHOLE	FLXPBA	FLXWHOLE
Calcium, mg	>= 800·0	957·1	935·6	474·7	1049·1	1150·1	946·4	797·4
Fat total, g	= 65·3	102·5	101·9	98·8	99·5	97·2	102·2	93·8
Fibre, g	>=25	19·4	32·1	40·0	26·5	30·1	25·7	30·4
Folate, ug	>=300	262·7	288·1	494·9	349·6	480·6	275·4	403·1
Iron, mg	>=9	10·0	14·2	19·2	10·4	13·0	12·1	13·5
Magnesium, mg	>=280	334·2	366·3	727·9	367·0	628·4	350·3	598·9
Phosphorus, mg	>=600	1588·8	1176·5	1279·0	1456·8	1669·2	1382·8	1535·0
Potassium, mg	>=3100	3067·9	2909·7	3581·2	2955·7	3346·1	2989·3	3453·6
Protein, g	=58·75	95·4	66·2	62·9	82·5	79·3	80·8	84·1
Riboflavin, mg	>=1·2	1·7	1·6	0·7	1·5	1·5	1·7	1·3
Selenium, ug	>=50	47·5	19·4	19·0	34·5	32·2	33·4	35·6
Sodium, mg	<=2400	1819·9	2374·4	1142·3	2069·9	1391·8	2097·2	1483·2
Monounsaturated fatty acids, g	<=115	34·1	35·5	33·0	32·0	30·3	34·8	31·3
Polyunsaturated fatty acids, g	>=13	13·7	22·4	27·9	17·3	19·0	18·1	19·9
Polyunsaturated fatty acids, g	<=26·1	13·7	22·4	27·9	17·3	19·0	18·1	19·9
Saturated fatty acids, g	<=26	42·2	27·4	24·9	36·2	36·6	34·8	31·7
Thiamine, mg	>=1·1	1·4	1·0	1·4	1·1	1·3	1·2	1·5
Vitamin B12, ug	>=2	5·7	1·8	0·0	2·9	2·9	3·7	3·0
Vitamin B6, mg	>=1·2	2·0	1·1	1·8	1·3	1·6	1·5	2·0
Vitamin C, mg	>=75	54·6	60·0	114·1	54·3	56·1	57·3	81·2
Vitamin D, ug	>=10	11·3	17·5	12·5	16·2	10·8	14·4	10·9
Vitamin E, mg	>=8	11·3	17·5	12·5	16·2	10·8	14·4	10·9
Vitamin K, ug	>=70	113·3	88·5	395·8	92·1	364·1	100·9	281·0
Zinc, mg	>=7	47·5	18·7	18·2	34·5	32·2	33·1	35·6

BAU *= Average Swedish Diet;* ***VGNPBA****= Vegan Diet, all ASFs replaced by PBAs;* ***VGTPBA****= Vegetarian Diet, meat& seafood replaced by respective PBAs;* ***FLXPBA****= 50% reduction of ASFs replaced by PBAs;* ***VGNWHOLE****= Vegan Diet, all ASFs replaced by WFs;* ***VGTWHOLE****= Vegetarian Diet, meat& seafood replaced by WFs;* ***FLXWHOLE*** *=50% reduction of ASFs replaced by WFs.*

A.2.2 Results from the uncertainty analysis

We conducted two types of sensitivity analysis for the nutritional performance, environmental impact and daily food expenditure of the different scenarios. In the first sensitivity analysis, we build the scenarios using an energy-based unit and paired the scenarios with impact per unit kcal instead per unit gram to account for potential strengths and limitations of the functional units. In the second sensitivity analysis, we accounted for the major uncertainties in each analysis by calculating the 25th and 75th percentiles in addition to the median values (50th percentiles). For the nutritional analysis, we calculated lower and upper values, 25th and 75th percentile impacts for each macro- and micronutrient from the Swedish National Food Agency database for the respective food items. In analysing environmental impacts, we assessed 25th and 75th percentile ranges for the PBAs to determine how sensitive food choices are to the environmental impact of the dietary scenario. And in the daily food expenditure analysis, we used the range of prices of food items extracted from the food retailer to calculate the 25th and 75th percentile values for the more aggregated food groups to provide a price range and better reflect individual consumer behaviour.

A.2.4.1 Nutritional Performance

Supplemental Figure 3: Nutritional performance of the respective dietary scenarios in alignment with the Recommended Dietary Allowances provided by the Nordic Nutrition Recommendations (0 = respective recommended nutrient level) **when using the energy-based functional unit**.

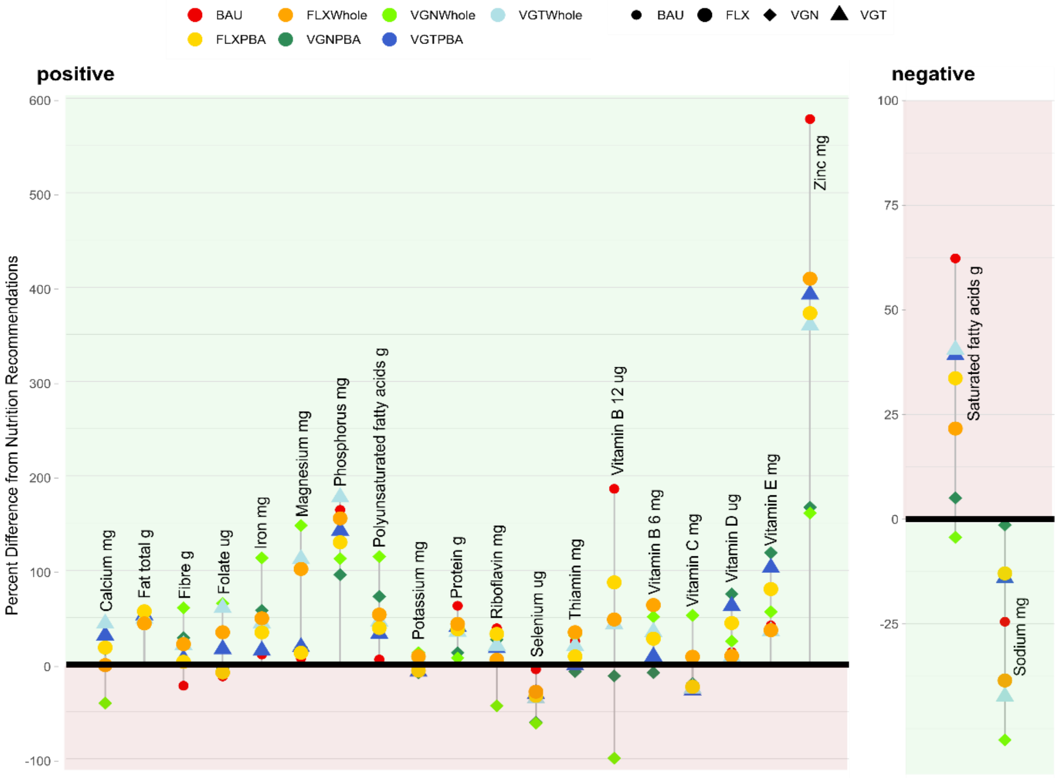

Green space (above 0 in 'positive' and below 0 in 'negative') indicates meeting or extending the recommended levels, red space (below 0 in 'positive' and above 0 in 'negative') indicates not meeting (or exceeding in nutrients to limit) recommended levels.* ***BAU* *= Average Swedish Diet;* ***VGNPBA*** *= Vegan Diet, all ASFs replaced by PBAs;* ***VGTPBA*** *= Vegetarian Diet, meat& seafood replaced by respective PBAs;* ***FLXPBA*** *= 50% reduction of ASFs replaced by PBAs;* ***VGNWHOLE*** *= Vegan Diet, all ASFs replaced by WFs;* ***VGTWHOLE*** *= Vegetarian Diet, meat& seafood replaced by WFs;* ***FLXWHOLE*** *=50% reduction of ASFs replaced by WFs.* Source data are provided as a Source Data file.

Supplemental Figure 4: Interquartile Ranges (IQR) of nutrient content within the different food groups. Using a mass-based functional unit.

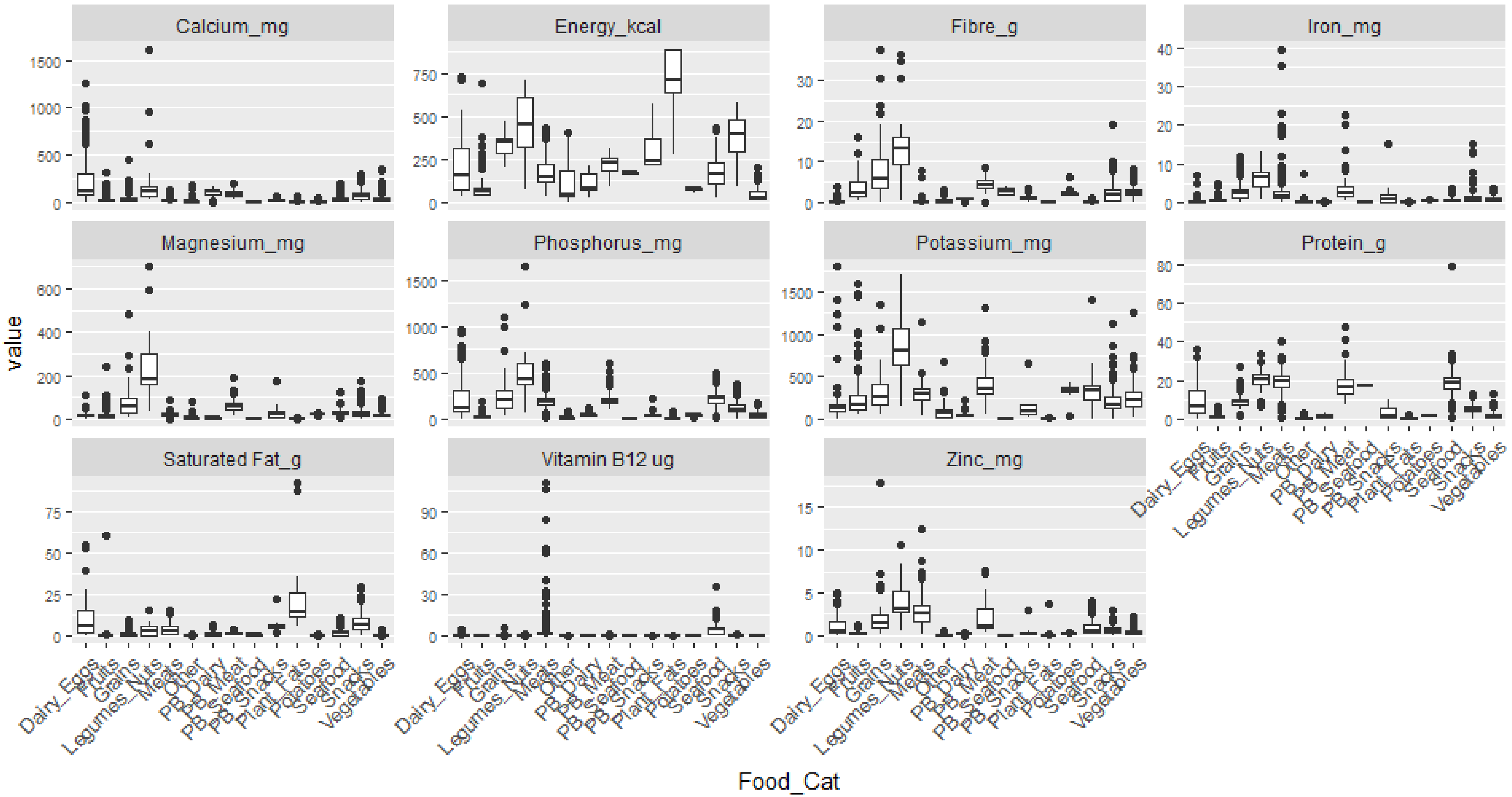

**The size of the box indicates the IQR. The vertical bar rule inside the box shows the median value for the nutritional content. The dots outside of the range of the whiskers in the box plot indicate outliers.*

A.2.4.2 Environmental impact of the different plant-based alternatives and legumes

Supplemental Figure 5: Environmental impact of the different dietary scenarios based on an energy functional unit.

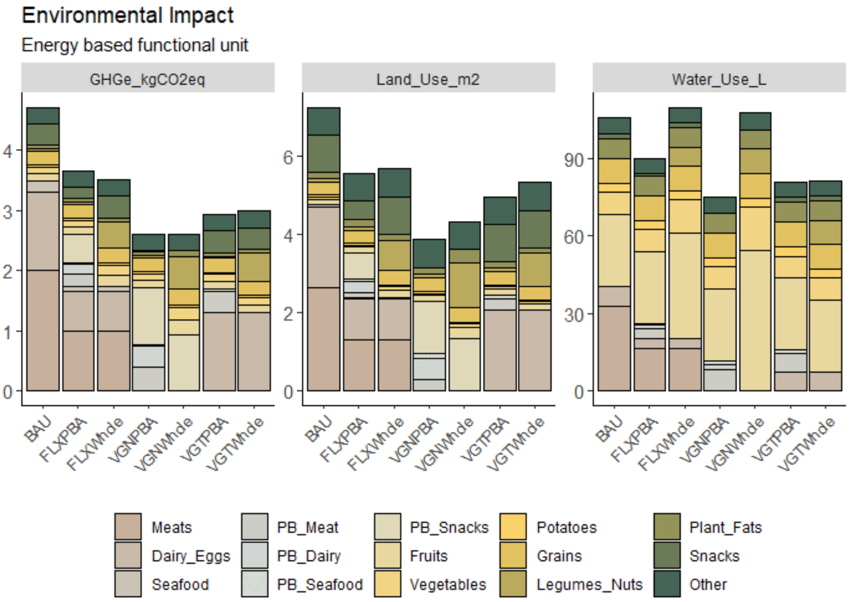

** Contribution by different food categories to impact categories. The category "other" includes sugar and sugar-based products, salt, soft drinks, coffee (roasted powder) and juice. BAU = Average Swedish Diet; VGNPBA= Vegan Diet, all ASFs replaced by PBAs; VGTPBA= Vegetarian Diet, meat& seafood replaced by respective (plant-based alternatives) PBAs; FLXPBA= 50% reduction of ASFs replaced by PBAs; VGNWHOLE= Vegan Diet, all ASFs replaced by (whole foods) WFs; VGTWHOLE= Vegetarian Diet, meat& seafood replaced by WFs; FLXWHOLE =50% reduction of ASFs replaced by WFs.* Source data are provided as a Source Data file.

Supplemental Figure 6: Environmental impact of PBAs and legumes by food group according to the functional unit. Values are medians with interquartile ranges (IQR).

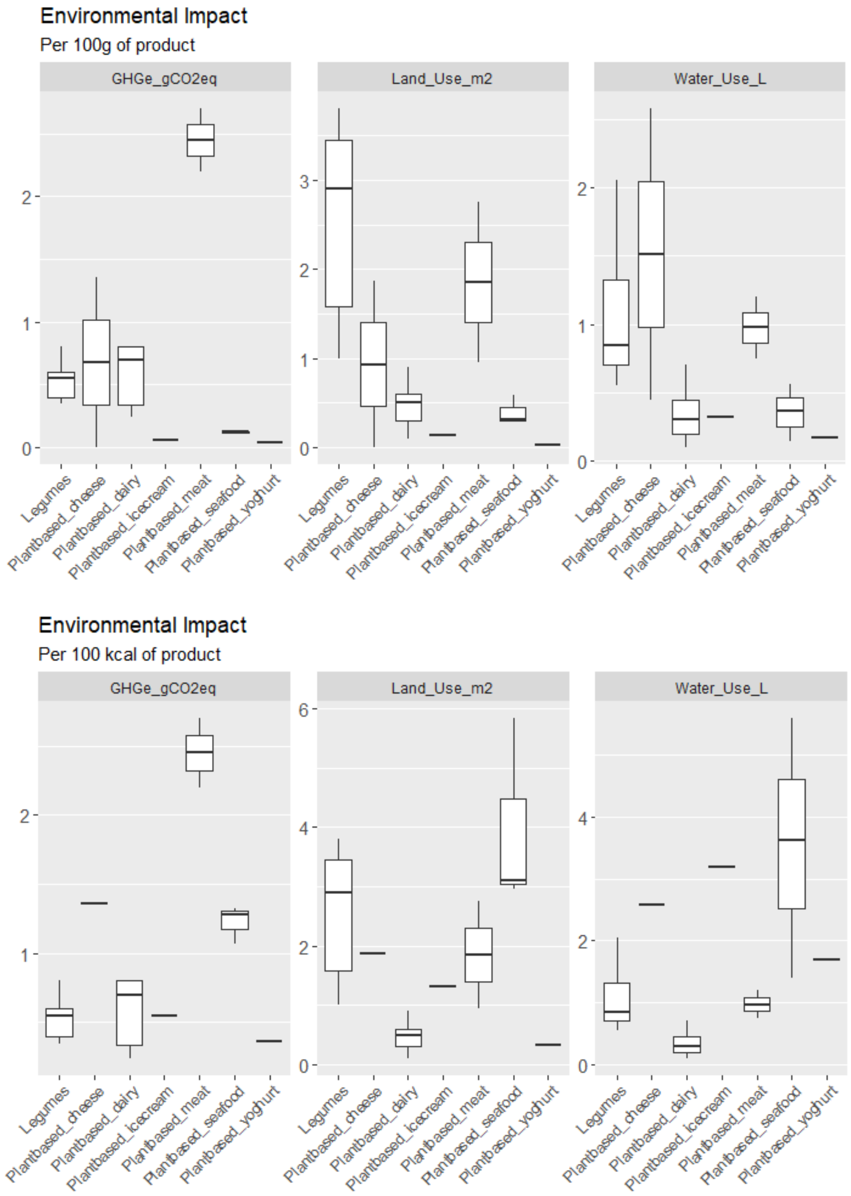

**The size of the box indicates the IQR. The bottom and top rule denote the bottom fifth and top fifth percentiles, respectively. The vertical bar rule inside the box shows the median value for the price value.*

A.2.4.3 Daily Food Expenditure

Supplemental Figure 7: Daily Food Expenditure (SEK per day) in June 2022 by dietary scenario and food group using an energy-based functional unit.

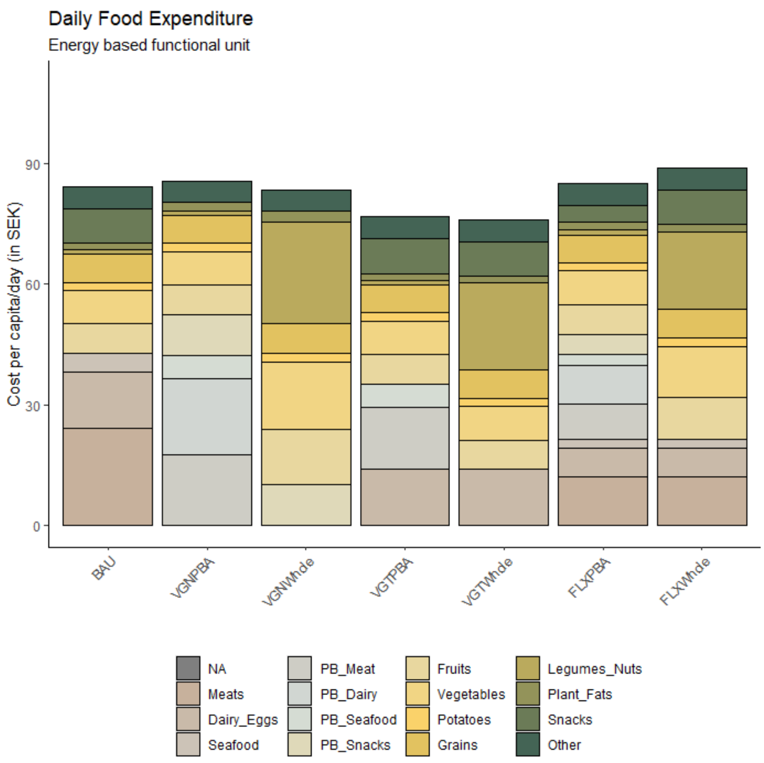

10 SEK = 0,9€ / 0.96 US$. Other includes sugar and sugar-based products, salt, soft drinks and juice. Data is presented as median values.* ***BAU* *= Average Swedish Diet;* ***VGNPBA*** *= Vegan Diet, all ASFs replaced by PBAs;* ***VGTPBA*** *= Vegetarian Diet, meat& seafood replaced by respective PBAs;* ***FLXPBA*** *= 50% reduction of ASFs replaced by PBAs;* ***VGNWHOLE*** *= Vegan Diet, all ASFs replaced by WFs;* ***VGTWHOLE*** *= Vegetarian Diet, meat& seafood replaced by WFs;* ***FLXWHOLE*** *=50% reduction of ASFs replaced by WFs.* Source data are provided as a Source Data file.

Supplemental Figure 8: Daily food expenditure ranges for the different scenarios. Values are medians with interquartile ranges (IQR).

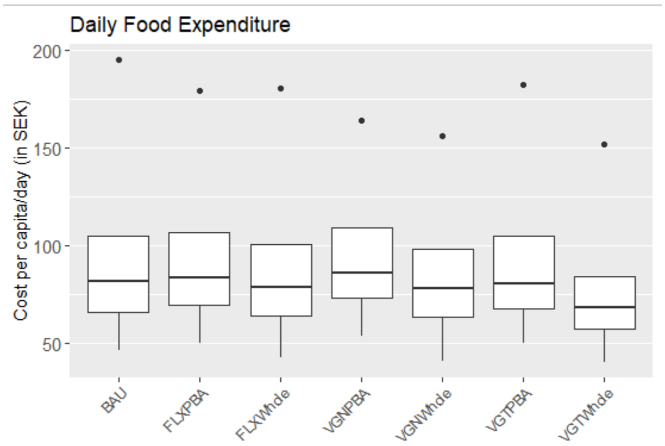

Supplementary References

1. Jordbruksverket. Livsmedelskonsumtion och näringsinnehåll. Uppgifter till och med 2019. Available at https://jordbruksverket.se/om-jordbruksverket/jordbruksverkets-officiella-statistik/jordbruksverkets-statistikrapporter/statistik/2020-12-09-livsmedelskonsumtion-och-naringsinnehall.--uppgifter-till-och-med-2019 (2019) .
2. Gustavsson, J., Cederberg, C., Sonesson, U., Otterdijk, R. V. & Meybeck, A. Global food losses and food waste: extent, causes and prevention (2011).
3. Martin, M. & Brandão, M. Evaluating the Environmental Consequences of Swedish Food Consumption and Dietary Choices. *Sustainability* **9**, 2227 (2017).
4. Springmann, M. *et al.* Health and nutritional aspects of sustainable diet strategies and their association with environmental impacts: a global modelling analysis with country-level detail. *Lancet Planet. Health* **2**, e451–e461 (2018).
5. Hornborg, S., Bergman, K. & Ziegler, F. Svensk Konsumtion av sjömat (2021).
6. Amcoff, E. *et al*. Riksmaten - vuxna 2010-11. Livsmedels- och näringsintag bland vuxna i Sverige. Available at https://www.livsmedelsverket.se/globalassets/matvanor-halsa-miljo/kostrad-matvanor/matvaneundersokningar/riksmaten_2010_20111.pdf (2012).
7. Willett, W. *et al.* Food in the Anthropocene: the EAT-Lancet Commission on healthy diets from sustainable food systems. *The Lancet* **393**, 447–492 (2019).
8. Livsmedelsverket. Energi, kalorier. Available at https://www.livsmedelsverket.se/livsmedel-och-innehall/naringsamne/energi-kalorier (2023).
9. Masset, G., Vieux, F. & Darmon, N. Which functional unit to identify sustainable foods? *Public Health Nutr.* **18**, 2488–2497 (2015).
10. Hallström, E., Davis, J., Woodhouse, A. & Sonesson, U. Using dietary quality scores to assess sustainability of food products and human diets: A systematic review. *Ecol. Indic.* **93**, 219–230 (2018).
11. Mertens, E. *et al.* Potential Impact of Meat Replacers on Nutrient Quality and Greenhouse Gas Emissions of Diets in Four European Countries. *Sustainability* **12**, 6838 (2020).

12. Costa, C. *et al.* Roadmap for achieving net-zero emissions in global food systems by 2050. *Sci. Rep.* **12**, 15064 (2022).
13. Livsmedelsverket. The Swedish Food Composition Database. Available at https://www.livsmedelsverket.se/en/food-and-content/naringsamnen/livsmedelsdatabasen.
14. Hooked Foods. Vegetarian and vegan seafood - Hooked Foods. Available at https://hookedfoods.com/products/.
15. Moberg, E., Karlsson Potter, H., Wood, A., Hansson, P.-A. & Röös, E. Benchmarking the Swedish Diet Relative to Global and National Environmental Targets—Identification of Indicator Limitations and Data Gaps. *Sustainability* **12**, 1407 (2020).
16. Karlsson Potter, H., Lundmark, L. & Röös, E. Environmental impact of plant-based foods–data collection for the development of a consumer guide for plant-based foods. (2020).
17. Karlsson Potter, H. & Röös, E. Multi-criteria evaluation of plant-based foods –use of environmental footprint and LCA data for consumer guidance. *J. Clean. Prod.* **280**, 124721 (2021).
18. Tidåker, P., Karlsson Potter, H., Carlsson, G. & Röös, E. Towards sustainable consumption of legumes: How origin, processing and transport affect the environmental impact of pulses. *Sustain. Prod. Consum.* **27**, 496–508 (2021).
19. Harrington, R. A., Adhikari, V., Rayner, M. & Scarborough, P. Nutrient composition databases in the age of big data: foodDB, a comprehensive, real-time database infrastructure. *BMJ Open* **9**, e026652 (2019).
20. Clark, M. *et al.* Estimating the environmental impacts of 57,000 food products. *Proc. Natl. Acad. Sci.* **119**, e2120584119 (2022).
21. Axfood. Willys – Swedens leading discount grocery chain. Available at https://www.axfood.com/about-axfood/the-axfood-family/willys/.
22. Nordic Council of Ministers. Nordic Nutrition Recommendations 2012: Integrating Nutrition and Physical Activity. Available at https://www.norden.org/en/publication/nordic-nutrition-recommendations-2012 (2014).

23. European Union. The EFSA Comprehensive European Food Consumption Database. Available at https://data.europa.eu/euodp/en/data/dataset/the-efsa-comprehensive-european-food-consumption-database (2018).
