# Supplementary material for: Sustainability benefits of transitioning from current diets to plant-based alternatives or whole-food diets in Sweden
Source: Nat Commun. 2024 Feb 1;15:951. doi: 10.1038/s41467-024-45328-6 (PMC10831109; doi:10.1038/s41467-024-45328-6)
Supplement: Supplementary file 3 — Description of additional supplementary files [file 41467_2024_45328_MOESM3_ESM.pdf]

Description of additional supplementary files

Supplementary data 1 : Dietary Scenarios

Description : Detailed overview on the dietary scenarios on a kcal and mass basis.

Supplementary data 2 : Product Database

Description : All product data input into the calculation including environmental impact, nutritional values, price data and source name of the food product data.

Supplementary data 3 : RDAs

Description : Overview of the Recommended Dietary Allowances by the Nordic Nutrition Recommendations that have been used to assess the relative nutritional adequacy of the dietary scenarios.

Supplementary data 4 : Results Nutr.Performance

Description : Detailed results displaying the proportional contribution of the respective food groups to macro-and micronutrient content of the dietary scenarios. The results are displayed on both a mass and energy functional unit.

Supplementary data 5 : Results Env. Impact

Description : Detailed results displaying the proportional contribution of the respective food groups to the environmental impact of the dietary scenarios using both a mass and energy-based functional unit.

Supplementary data 6 : Results Price

Description : Detailed results displaying the proportional contribution of the respective food groups to the daily food expenditure of the dietary scenarios. Results are displayed on both a mass and energy functional unit.
